# A Few Practical Hints on Sponge Tents and Their Uses

**Published:** 1872-03

**Authors:** Philip Adolphus

**Affiliations:** 318 West Madison Street; Chicago


Article III.—*A Few Practical Hints on Sponge Tents and their Uses.* By Philip Adolphus, M.D., Chicago.

(second paper.)

Having in a previous paper spoken of the sponge tent in connection with diagnosis, the writer will pass in review the different diseases, in the treatment of which the sponge tent is useful.

The curative influence of the sponge tent manifests itself in all diseases in which it is employed, by dilatation, pressure and absorption. Pressure produces dilatation, and is an efficient means of stimulating absorption; thus removing deposit. Absorption excites the absorbent vessels to increased action, and therefore causes a more or less gradual, painless loss of texture before slowly increasing pressure. The sponge tent thus is the agent to remove the redundant morbid material. It is a curative agent, when it does this whilst exciting the arterial and capillary vessels little, if at all; but when it is intense enough in its action to arouse the nerves to irritation, or the bloodvessels to an assumption of inflammation, it produces cellulitis, peritonitis, hæmatocele, or intense pain in the pelvic organs; irritation in distant parts, either by sympathy of function or by continuity of nervous and other tissues. The many accidents mentioned by authors, after the use of the sponge tent, are thus apparently accounted for. But what experienced practitioner in the treatment of uterine complaints does not know, that symptoms of local and constitutional irritation are more or less constantly present, or supervene, in all grave uterine complaints, from the slightest causes, and often from no appreciable cause? Have we not perversion of function pervading the whole system? The impaired strength, the anxious countenance, restlessness, disturbed and unrefreshing sleep; the cold and pale surface, disinclination for food, clammy skin, confined bowels, and scanty urine: or again, the profuse perspiration, diarrhœa, the copious diureses and the vast array of neuralgic manifestations. How often are we called to a patient, suffering acute pain of the abdomen, simulating peritonitis or hæmatocele; yet the most searching local manipulation cannot detect these diseases. A dose of morphia, a few leeches, a mustard plaster, a hot poultice, or a local application of chloroform, will dispel the attack, the patient will be found relieved at our next visit, and this same state of affairs will repeat itself periodically and intermittingly.

Nevertheless, despite these apparently causeless attacks, it behooves us to be careful in the use of the sponge tent, and, besides using the precautions so minutely set forth in the preceding paper, "on the sponge tent as regards diagnosis," it is essential, that no indiscreet haste be indulged in the treatment of uterine diseases, with this agent. The treatment is one of months, not days; gradual, interrupted. The pressure requires to be neither great nor constant; and only by an ever-present remembrance of these principles will treatment be conducted so prudently and patiently as to avoid these ill results to which inordinate pressure by the sponge tent is liable.

It is advisable to precede the remedial use of the sponge tent by local bloodletting or scarification; especially in ailments in which the ovaries are implicated—and they are always involved in grave affections of the uterus. These local depletions should be undertaken a few days before the expected menstrual period.

The tent should not be large enough to produce irritation when placed in situ; it should not impinge on the fundus uteri. It is to be inserted with the same precautions as advised in a previous paper; the time of insertion should be about ten days from the completion of the last flow. A suppository of cotton wool saturated with glycerine is placed immediately over the os uteri, in order to receive the discharge and absorb and neutralize its offensiveness. Let it be borne in mind, that whilst it is not expedient to leave the tent longer than twenty-four hours in the uterus, when its neck is expanded as a means of diagnosis, the tent used as a remedial agent is left in the uterus as long as the patient is not inconvenienced by it. Examine the abdomen daily by pressure; ask the patient whether any pain or irritation is experienced; retract the perineum with two fingers of the left hand, remove the suppository, insert the speculum, press the forceps armed with moist cotton wool against the sponge tent emerging from the os uteri; thereby removing the discharge, and replace the removed suppository by another saturated with glycerine; repeat this daily until the tent is finally removed.

It is not generally expedient to leave the tent longer in situ than 72 hours; for then, despite the presence of the glycerine suppository, the patient will be annoyed with the fetor of the tent. On its removal cleanse the uterus with tepid water by means of a double current catheter, and insert a pledget of cotton wool, saturated with equal parts of compound tincture of iodine and fluid extract of aconite, into the uterus by means of Emmet's applicator. This is left in situ, a suppository charged with glycerine is laid over the os, and the patient is enjoined to remain as quiet as possible.

Therefore the rule holds good to remove the tent whenever it causes irritation; to leave it, when it does not do so, until the fetor it causes becomes annoying to the patient.

Constitutional and hygienic treatment, together with hot vaginal irrigation, as recommended by Emmet, is essential to the successful result of the cure.

In all cases of chronic enlargement of the womb, so-called general hypertrophy, subinvolution, etc., complicated or not with versions or prolapse, the sponge tent, in certain stages of the disease, used at stated periods, is an efficient aid in its treatment.

We find these cases in persons whose pregnancies have succeeded each other rapidly, and who have been subject to abortions. All the tissues of the womb may be found enlarged or only the cervical or vaginal portion; its connective tissue has increased greatly, not necessarily caused by inflammation, though this may be its result. We have congestion, to which is added that periodical active hyperæmia caused by the menstrual molumen.

Engorgement of the blood vessels of the mucous membrane is present, accompanied by abnormal secretion; we have swelling, succulence of the tissues, and copious generation of young cells (Niemeyer). If this congestion does not relieve itself by hæmorrhage, more persistent and active excitement ensues, and the discharge becomes muco-purulent, then purulent, chronic inflammation of the tissues is added (chronic catarrh) to the already existing proliferation of the connective tissue.

On bi-manual palpation the uterus is found enlarged, painful on pressure; the finger introduced into the rectum ascertains the uterus pressing on this organ, heavy, perhaps retroflexed or prolapsed. The ovaries are nearly always painful, and the usual nervous phenomena generally present, to which may be added those of pressure; menstruation is painful.

Now leechings around the margin of the anus, on the perineum, around the os uteri; internal scarifications of the uterus and its cervix; external scarifications of its vaginal portion are indicated, and will be followed by beneficial results.

Therefore in the cases above described, local depletion should always precede the sponge tent or any other local application. The first three or four leechings may be practiced at any time, to relieve pressing symptoms; afterwards, they should be timed a few days previously to the menstrual flow.

Emmet, who has been for years using tents in just such cases, does not precede their use by local depletion; his vast experience surely would entitle imitation; yet the writer must candidly acknowledge that in his hands, previously to his using local depletory measures, cellulitis had occurred in several cases.

This is referred to here, not for the purpose of calling the expediency of Emmet's treatment in question, for its success in his hands is the best argument for its continuance; but rather to illustrate that a specific treatment in the hands of one man, by reason of greater experience and nicer discrimination, may be safe and efficient, which, repeated by others, proves a source of injury to their patients. The sponge tent in these cases, by the alterative influence of pressure on the uterine surfaces, produces absorption, softening and depletion, not merely of the neck of the uterus but of its body.

When, in consequence of hyperæmia, the uterus becomes enlarged by diffuse proliferation of its connective tissue, hypersecretion of the glands is induced; so-called nabothian vesicles are developed, the glands degenerate and become cysts; polypi thus originate, and, on account of their vascularity, often cause great hæmorrhage. These morbid growths when pediculated are of course removed by the forceps or scissors, after proper dilatation; but we find others, non-pediculated, imbedded in the mucous membrane, sessile. The insertion of a sponge tent, in the manner described in a previous paper, will effect their absorption, together with the restoration of the diseased mucous membrane, which caused their appearance.

The sponge tent is also beneficial in the diseased condition called by authors, endocervicitis and cervical endometritis. Our distinguished townsman, Dr. Byford, guided by the same indications, uses slippery elm bougies with great success in these cases. They are made of the same shape and length as the sponge tent, by means of a pocket-knife and sand-paper; introduced by the dressing forceps, moistened, and bent to the requisite shape of the uterine canal. They are permitted to remain for twelve hours unless sooner removed by pain, and are applied once every twelve days. The patient removes the tent by means of an attached string, which emerges from the vulva.

Dr. Thomas employs medicated sponge tents in this disease with decided success, and speaks highly of their use. The sponge is soaked in solutions of carbolic acid, lead, zinc, copper and iron; is then dried and prepared in the usual manner. They are antiseptic, and communicate to the mucous membrane the alterative qualities of the ingredients.

The writer applies pledgets of cotton wool soaked in various solutions, which he inserts by means of Emmet's applicator in the os and body of the uterus, and leaves them in situ. They will be found in the vagina in the course of twelve hours. The alterative influence of gentle pressure, together with the effect of the medication exercised by the drug in which the lint is steeped, are the remedial influences which he intends to apply to the diseased mucous membrane in this class of cases.

Cases where the mucous membrane of the neck and body is succulent, uneven and papillary, and the elevated and circumscribed granulations are red and granular, giving rise to great hæmorrhage, and which Recamier treated by scraping them with the curette, and to which Sims applied chromic acid, are well adapted to the treatment of the sponge tent; which, by pressure and distention, effects a cure. This is an old and well approved mode of treatment introduced by Simpson.

When are sponge tents indicated in dysmenorrhœa? Dysmenorrhœa is merely a symptom which presents itself in a great variety of cases. Symptoms of pressure, distention or retention may exist; these being relieved, the case is not cured, but a search for the cause is required. It is not sufficient merely to relieve pain, the gynæcologist ascertains the cause of the symptoms, and consequently treats the case understandingly.

All cases of dysmenorrhœa should at first be treated empirically; that is, urgent indications should be met by an appropriate treatment during the menstrual nisus. Should we succeed in witnessing a painless natural flow, our duty has been performed. If otherwise, a thorough examination is essential. The following classes of cases require no examination :

Where, according to Scanzoni, the unequal distribution of blood which characterizes anæmia, is the cause of dysmenorrhœa ;

Where the amount of flow being too great, it accumulates within the uterus, distending it, thereby causing delay and exciting it to pain ;

Cases, occurring in virgins, which are characterized by phenomena of congestion and nervous irritation of the uterus and ovaries.

*The principal indication in the mechanical treatment of all diseases which are complicated with dysmenorrhœa, is to induce the proper patency of the uterine canal.* With few exceptions, the treatment should be conducted during the interval, when the monthly hyperæmia of the pelvic organs is in abeyance.

In all cases, inflammatory conditions must be first subdued. If fibroids and polypi are obstructive by their position, they should be removed.

Spasm of the cervical canal is oftener met than is supposed ; for it is partly muscular, and the cervix and os internum have muscular fibres entering into their structure ; therefore spasm is often produced on the introduction of instruments. Here, after local depletion, the bougie of Tilt, the slippery elm tent of Byford, and the small sponge or laminaria tent, answer an admirable purpose and effect a cure.

The tent and other dilating agents hold, however, a subordinate part in the mechanical treatment of dysmenorrhœa. For strictures induced by malpositions of the uterus, and hypertrophied and otherwise diseased cervix, are best treated by incisions.

Dilating measures complete the restoration of the patency of the canal, and may from time to time be used. It is always proper to introduce a tent for a few hours previously to the succeeding menstrual flow in cases where incisions have been previously employed.

The employment of a series of sponge tents is indicated in cases of amenorrhœa, caused by infantile or rudimentary uterus. Their employment is followed by the happiest results. A series of graduated tents should be used, commencing with the smallest.

General treatment is at the same time employed. These local measures, together with monthly leechings, are instituted, not for the purpose of producing absorption or curing congestion, but rather to invite and develop local stimulation and distension.

Finally; when, in cases of abortion, hæmorrhage is great and the loss of the embryo inevitable, the introduction of a sponge tent into the cervix is more effectual than the vaginal tampon. It induces uterine contractions, dilates the cervix, and restrains hæmorrhage. Should the embryo have escaped, the secundines be retained, and hæmorrhage be profuse, the introduction of a sponge tent into the cervix again restrains hæmorrhage, prepares the way for the introduction of the placental forceps or finger, and avoids the use of the annoying and filthy rag tampon, in the removal of which the patient experiences so much pain, and the practitioner, by reason of the insupportable fetor, so much annoyance. The suppository of cotton charged with glycerine, inserted at the same time into the vagina, will, by its cleansing, purifying and lubricating qualities, obviate fetor, pain and uncleanliness. No one who has once tried the sponge tent and glycerine plug, in cases of abortions, will again deny himself the valuable assistance it confers. To Simpson we are indebted for the first suggestion, whilst Sims has the merit of having introduced glycerine in uterine surgery.

318 West Madison Street.

